# Hyperspectral imaging for simultaneous measurements of two FRET biosensors in pancreatic β-cells

**DOI:** 10.1371/journal.pone.0188789

**Published:** 2017-12-06

**Authors:** Amicia D. Elliott, Noah Bedard, Alessandro Ustione, Michelle A. Baird, Michael W. Davidson, Tomasz Tkaczyk, David W. Piston

**Affiliations:** 1 National Institute of General Medical Sciences, Bethesda, MD, United States of America; 2 Rice University, Bioengineering, Houston, TX, United States of America; 3 Washington University in St. Louis, St. Louis, MO, United States of America; 4 The Florida State University, National High Magnetic Field Laboratory, Tallahassee, FL, United States of America; University of Michigan, UNITED STATES

## Abstract

Fluorescent protein (FP) biosensors based on Förster resonance energy transfer (FRET) are commonly used to study molecular processes in living cells. There are FP-FRET biosensors for many cellular molecules, but it remains difficult to perform simultaneous measurements of multiple biosensors. The overlapping emission spectra of the commonly used FPs, including CFP/YFP and GFP/RFP make dual FRET measurements challenging. In addition, a snapshot imaging modality is required for simultaneous imaging. The Image Mapping Spectrometer (IMS) is a snapshot hyperspectral imaging system that collects high resolution spectral data and can be used to overcome these challenges. We have previously demonstrated the IMS’s capabilities for simultaneously imaging GFP and CFP/YFP-based biosensors in pancreatic β-cells. Here, we demonstrate a further capability of the IMS to image simultaneously two FRET biosensors with a single excitation band, one for cAMP and the other for Caspase-3. We use these measurements to measure simultaneously cAMP signaling and Caspase-3 activation in pancreatic β-cells during oxidative stress and hyperglycemia, which are essential components in the pathology of diabetes.

## Introduction

Spectral imaging techniques can be powerful tools for fluorescent protein (FP) imaging of living cells. Biosensors that rely on intramolecular Förster resonance energy transfer (FRET) are commonly used as probes for biological function [1]. Many FRET-based biosensors contain a cyan fluorescent protein (CFP) variant donor and a yellow fluorescent protein (YFP) variant acceptor [2]. Despite the creation of an efficient green/red FRET pair [3], dual-FRET remains challenging due to the overlapping emission spectra of the FPs. Fluorescence lifetime imaging microscopy (FLIM) has been used for dual-FRET applications [4], but FLIM offers limited temporal resolution (> 10 sec), so it is too slow to reveal relationships between many sequential signaling pathways and rapid changes in cellular response to stimuli. Hyperspectral methods allow for the collection of the entire fluorescence emission spectrum at each pixel in an image, thus providing a means of quantitative imaging of multiple probes in a single experiment [5,6]. In principle, these approaches are well-suited for dual-FRET imaging, but most hyperspectral imaging methods rely on sequential image acquisitions that are incompatible with truly simultaneous multi-color imaging. Furthermore, the data analysis is challenging, as quantitation of hyperspectral FRET experiments relies upon reference spectra, high quality data and spectral resolution [7]. Finally, most multispectral imaging systems use sequential scanning either in space or wavelength, which requires increased illumination times [8,9]. Thus, these methods are limited in spectral and/or temporal resolution and lack sufficient dynamic range for quantifying the small changes measured by most FRET-based biosensors.

An alternative hyperspectral imaging approach that addresses many of these challenges is the Image Mapping Spectrometer (IMS). The IMS is a camera-based snapshot hyperspectral imaging system that allows rapid collection of high-resolution spatial and spectral data in a single snapshot with a single excitation source. When coupled to a wide-field microscope, the IMS does not require sequential scanning in space or wavelength and has an image acquisition rate tha tis limited only be the camera speed or signal intensity. In the system described here, the camera used provides a large field-of-view (225 x 240 μm) with acquisition rates up to 7.2 frames per second. The IMS uses an image mapper that spatially distributes neighboring pixels in the intermediate image to create blank regions in the final image. The spectral content of each pixel is then dispersed into these blank regions so that the final image is mapped onto a 2D detector array where each pixel represents a unique x, y, λ value. This method allows parallel measurement of a sample’s x, y, λ datacube without scanning, thus collecting the whole datacube in a single snapshot with high optical throughput [10]. The widefield IMS used here collects data within a spectral window from 450 nm to 650 nm with ~4 nm sampling interval, which is sufficient for differentiating commonly overlapping fluorophores, such as CFP, GFP, and YFP. We have previously demonstrated that the IMS coupled to a widefield microscope is useful for simultaneously studying time-resolved cAMP and Ca^2+^ signaling dynamics in pancreatic β-cells [11]. This was accomplished with biosensors for cAMP, a cyan/yellow FRET sensor (^T^-Epac-^VV^) [12], and a cpGFP-based Ca^2+^ sensor [13] with overlapping spectra. This work verified that the IMS can effectively separate spectra with differences greater than two spectral bins (in this case > 8 nm), as expected from Nyquist sampling theory. Another recent configuration of the IMS coupled to a smaller but faster sCMOS camera and a light-sheet microscope demonstrated the measurement of Ca^2+^ and Zn^2+^ in different populations of cells in intact tissue [14].

To test the IMS for dual-FRET imaging in the widefield configuration, we investigated the effects of oxidative stress and hyperglycemia on cAMP signaling while monitoring Caspase-3-mediated apoptosis in cultured MIN6 pancreatic β-cells. These experiments used an established cAMP biosensor [12], and a newly prepared Caspase-3 cleavable biosensor (GRSCAT) with the green/red FRET pair mClover and mRuby2 [3]. Dysregulated β-cell apoptosis contributes to the development of type 2 diabetes and limits the effectiveness of islet transplantation therapy for patients with type 1 diabetes [15–17]. However, few tools are available to measure the process of apoptosis in live-cell experiments, which limits the ability to determine the cellular processes that are important for inducing β-cell death. The GRSCAT sensor presented here is an intramolecular FRET-based probe built with a Caspase-3 cleavage site between the mClover and mRuby2 FPs [3], and provides a live measure of apoptosis induction.

The generation of reactive oxygen species (ROS) is an important route by which β-cell apoptosis is stimulated during diabetes, though little is known about the molecular mechanism that drives it. Several G-protein coupled receptors are critical regulators of β-cell function and are known to proceed via cAMP, so we took advantage of the sensors that we had available to probe this signaling pathway. It has been shown that NADPH oxidase reduces cAMP/PKA signaling secondarily to ROS generation and reduces insulin secretion, suggesting that cAMP signaling is redox-responsive [18]. Another study showed that increasing cAMP levels led to increases in c-myc expression that drove Caspase-3 activation and apoptosis, though in the absence of oxidative stress [19]. These data suggest a possible role for cAMP signaling in apoptosis, but provide different mechanistic explanations for it depending on the redox state of the β-cells. Thus, the molecular mechanisms underlying ROS generation during oxidative stress and hyperglycemia and the subsequent effect on apoptosis remain unclear.

Here, we develop a dual-FRET approach to measure simultaneously cAMP and Caspase-3 activity, using a novel Caspase-3 biosensor. We validate the quantitative separation of these two signals using a single excitation band and hyper-spectral imaging by IMS. We show the use of this new quantitative dual-FRET IMS approach to measure functional dynamics in MIN6 pancreatic β-cells. We treat the cells with high glucose and/or hydrogen peroxide to dynamically interrogate the relationship between cAMP signaling and mitochondria-mediated apoptosis, via Caspase-3 activation. By selectively inducing hyperglycemia and oxidative stress, we measured the relationship between these states in an effort to better understand cellular signaling mechanisms in β-cell death.

## Materials and methods

### Biosensors

A green-red FRET pair version of the SCAT3.1 biosensor [20] was created by direct replacement of CFP with mClover [5] and YFP with mRuby2 [3]. The resulting green-red FRET biosensor is referred to as green-red SCAT, or GRSCAT. The cyan/yellow FRET cAMP biosensor (^T^-Epac-^VV^) from the Jalink lab, mTurqDel-EPAC(dDEPCD)-cp173Venus(d)-Venus(d)(H74) was used as published with no further modifications [12]. Both sensors respond to increases in target molecules (cAMP and Caspase-3) with a decrease in FRET ratio.

### Cellular sample preparation

MIN6 cells (generous gift of R. Stein, also available from AddexBio, San Diego, CA) were transiently transfected with plasmid DNA encoding GRSCAT, and/or ^T^-Epac-^VV^. Transfection was accomplished using Lipofectamine2000 (Invitrogen, Carlsbad, CA) reagent according to the manufacturer's instructions. Cells were seeded onto coverglass-bottomed dishes (Mat-Tek) and cultured as previously described [21] for microscopy studies in DMEM with 11 mM glucose. Before imaging, cells were switched to the glucose concentration for imaging (5 mM or 20 mM) for three hours prior to imaging. Oxidative stress was induced by replacing the media with similar media containing 30% hydrogen peroxide prior to imaging. cAMP was increased with 50 μM Forskolin (Fsk) and 100 μM IBMX. Caspase-3 was stimulated independently of cAMP with 1 μM Staurosporine (STS).

### Time-lapse hyperspectral imaging

Hyperspectral fluorescence imaging experiments were performed on a Nikon inverted microscope TE300 with a 40×/1.0 CFI Plan Apochromat oil objective. The final pixel size in the IMS image corresponds to an object size of 0.7 μm, and an image size of 320 x 340 pixels. The cells were placed in extracellular-like buffer (140 mM NaCl, 5 mM KCl, 1 mM MgCl_2_, 5 mM glucose and 20 mM Hepes, pH 7.4), and epi-illuminated by a 75W Xenon lamp. A Chroma filter set (ex: BP 436/20, bs: DCLP455, em: HQ470LP) was used to separate fluorescence emission from excitation light. Both FRET pairs were excited by illumination through this single 436/20 bandpass filter. The intermediate image formed at the microscope side image port is directed to the entrance port of the IMS. The hyperspectral images were captured by the IMS and processed in MatLab for remapping and linear spectral unmixing [22], which separated signals from mTurquoise, cpVenus-Venus, mClover, and mRuby2 based on their spectral profiles and generated time-lapsed intensity changes for all expressed biosensors. Rolling ball background subtraction (radius 50 pixels) and fluorescence intensity measurement were performed using ImageJ, and GraphPad Prism was used for statistics. Data are normalized to control samples that were imaged at 5mM glucose.

## Results

### Spectral unmixing of two FRET biosensors

**Fig 1:**
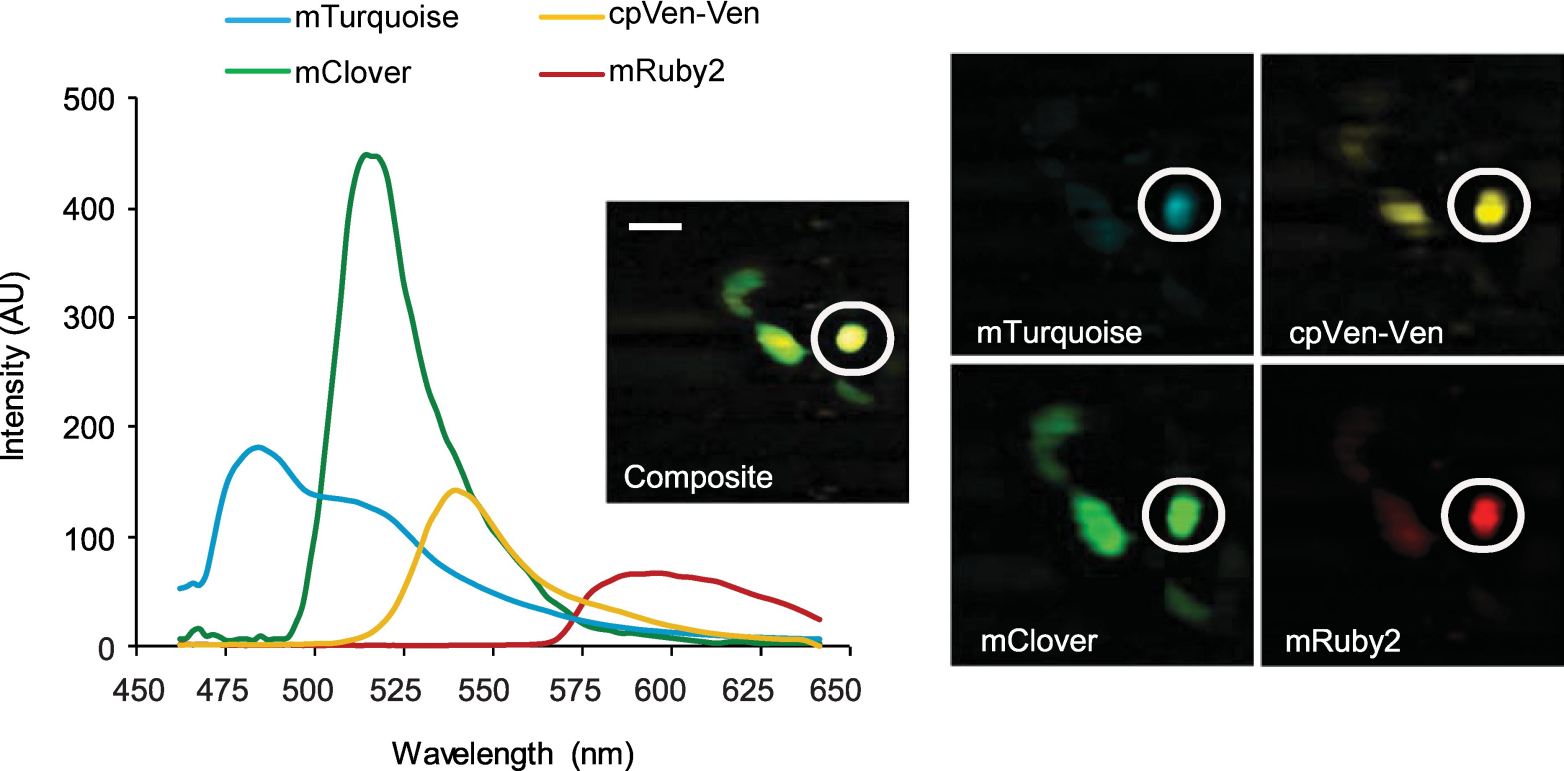
Linear unmixing two FRET biosensors. Raw intensities for the unmixed components of the FRET sensors ^T^-Epac-^VV^ and GRSCAT from the circled cell are shown (left) with the RGB images of the same FOV (right) Scale bar is 20 μm.

IMS-widefield microscopy provides a platform for rapid, simultaneous, multi-color live cell imaging. We co-expressed cAMP and Caspase-3 biosensors in MIN6 cultured pancreatic β-cells, yielding a four-color sample that offers the opportunity to study cellular signaling dynamics during hyperglycemia and oxidative stress. Reference spectra were collected from cells expressing individual FP components (mClover, mRuby2, mTurquoise, and mVenus-mVenus constructs) and used for unmixing [22]. Fig 1 shows a representative sample of cells expressing both FRET biosensors after linear unmixing. We previously demonstrated that the IMS system reliably separates the two donor FPs, mTurquoise and mClover [11], so we now wanted to show that the two FRET ratios could be deconvolved by the IMS as well.

**Fig 2:**
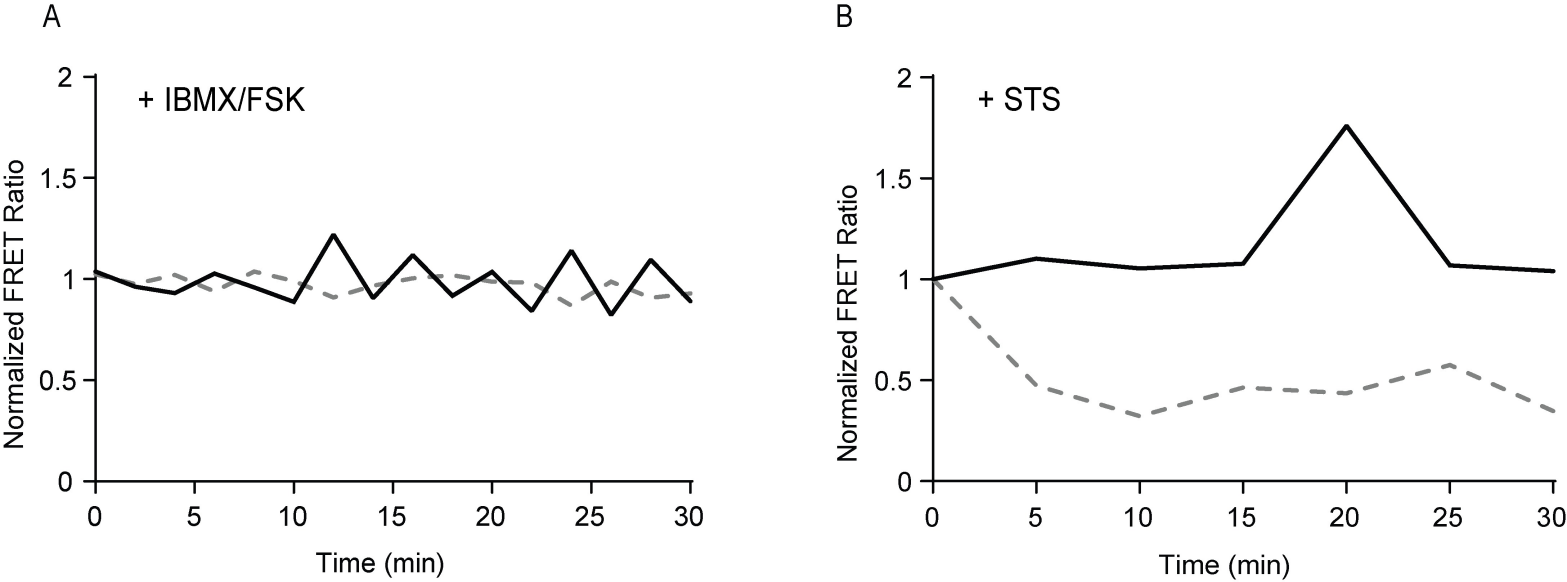
Dual-FRET biosensor isolation by selective stimulation. Time course of Caspase-3 activation (dotted gray lines) and cAMP (solid black lines) after stimulation with (A) IBMX/forskolin or (B) STS normalized to 5 mM glucose controls.

As a first test of the functionality of the cellular system expressing both biosensors, we treated co-expressing cells with chemicals that are expected to affect only the FRET changes from a single biosensor. To functionally isolate the cAMP biosensor response, cells expressing both the cAMP sensor and the Caspase-3 GRSCAT sensor were treated with IBMX/forskolin to increase cAMP amplitudes. This treatment is expected to change cAMP levels with minimal perturbations to Caspase-3 activity. As shown in Fig 2, IBMX/forskolin exposure (added at t = 0) increased cAMP and high glucose elicited oscillations in the ^T^-Epac-^VV^ cAMP sensor FRET ratio in 30 minutes with no significant change in the GRSCAT FRET ratio. The fungal toxin staurosporine (STS), which has been shown to directly and quickly activate Caspase-3 [23], was used to stimulate Caspase-3 in co-expressing cells immediately prior to imaging. Similar to the IBMX/forskolin experiments, we expected STS to lead to changes in the GRSCAT sensor with little change in the cAMP biosensor. As expected, we observed a 27.3% ± 3.2 decrease in the GRSCAT FRET ratio in 30 minutes following STS treatment with no significant change in the cAMP sensor FRET ratio (Fig 2), as measured by area under the curve from 4–10 cells per experiment.

### Dual-FRET imaging shows anti-correlated cAMP and Caspase-3 activity during oxidative stress and hyperglycemia

**Fig 3:**
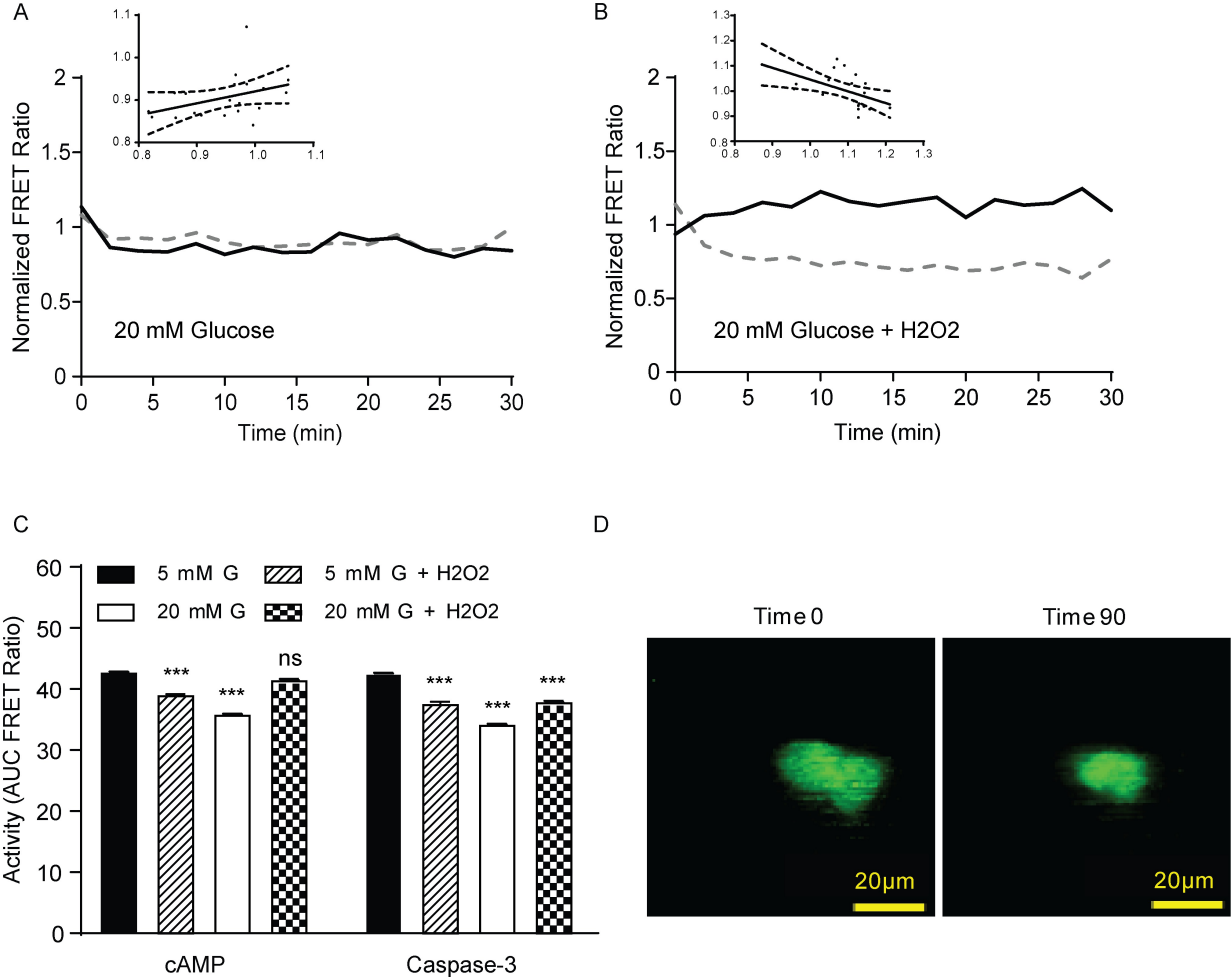
Role of oxidative stress in Caspase-3 activation and cAMP generation during hyperglycemia. (A) Time course of cAMP (solid black lines) or Caspase-3 activation (dotted black lines) for co-transfected cells treated with 20 mM glucose normalized to 5 mM glucose controls. (B) As in (A) but with the addition of H_2_O_2_. Insets for both (A) and (B) are linear correlations with 95% confidence intervals (x-axis is cAMP FRET ratio and y-axis is Caspase-3 FRET ratio). (C) Area under the curve for cAMP and Caspase-3 FRET measurements. These data are from one representative cell out of 3 different dishes, each dish had N = 4–20 cells. (D) IMS images of cells treated with high glucose and H_2_O_2_ at 0 and 90 minutes after imaging. p values were determined by ANOVA. *** indicates p < 0.001 compared to 5 mM glucose controls for each sensor.

To test the efficacy of the dual-FRET IMS approach to measure changes in cellular biology, we examined cAMP and Caspase-3 activation in hyperglycemic MIN6 cultured β-cells with or without inducing oxidative stress. Cells were cultured for 36–72 hours post-transfection with high glucose (11 mM). On the day of experiments, the cells were incubated for several hours with a high glucose concentration (20 mM) to simulate hyperglycemia prior to H_2_O_2_ addition to induce oxidative damage. Some samples were maintained at 20 mM glucose alone without additional H_2_O_2_, and separate control samples were incubated in parallel with 5 mM glucose. H_2_O_2_ was added to the dishes prior to imaging and baseline values were collected for the first two minutes of data collection. Consistent with expectations for high glucose, we find that cells pre-treated with 20 mM glucose displayed a 16.5% ± 0.54 increase in cAMP and a 20.9% ± 2.1 increase in Caspase-3 activity over low glucose controls (Fig 3), indicated by the decrease in area under the curve. Interestingly, under the additional condition of oxidative stress, cAMP levels were not significantly elevated over low-glucose controls (2.4% ± 3.8 increase), while Caspase-3 activity still increased significantly (15.8% ± 5.0). A representative single cell trace is shown in Fig 3, and the average over all cells measured under these conditions is shown in S1 Fig. To look at the relationship between the cAMP and Caspase-3 dynamics during oxidative stress, we performed a correlation analysis of the time-resolved FRET ratios. In the presences of high glucose alone without additional oxidative stress, we measured a non-significant positive correlation between cAMP and Caspase-3 (Fig 3; Pearson coefficient 0.3793, p = 0.0991). However, the addition of oxidative stress led to a significant inverse correlation between cAMP and Caspase-3 (Fig 3; Pearson coefficient -0.5384, p = 0.0143). Taken together, these data suggest that Caspase-3 activity and cAMP signaling are independent of each other but inversely correlated in a redox-dependent manner during oxidative stress and hyperglycemia. Characteristic cell shrinking and blebbing could be observed in the cells where Caspase-3 activity was high (Fig 3).

## Discussion

The success of previous experiments using a widefield-coupled IMS to resolve spectrally three fluorescent proteins during live cell imaging led us to test this system for simultaneous data collection from two FRET-based biosensors with overlapping spectra. We utilized this system to demonstrate simultaneous dual-FRET imaging with a single excitation. The data show minimal measured cross-talk between the FP components for the application of cAMP signaling and oxidative-stress induced Caspase-3 activation in pancreatic β-cells. These simultaneous dual-FRET (four color) experiments were made possible using hyperspectral imaging combined with a newly prepared green/red Caspase-3 cleavable FRET sensor and a previously-described cyan/yellow cAMP FRET sensor.

The results presented here demonstrate the ability of the IMS approach to address complex biological questions with high sensitivity along with excellent spatial and spectral resolution. The widefield-IMS configuration offers a large field-of-view, allowing for the visualization and measurement of many cells per experiment in a high-throughput format. The IMS methodology provides a sensitive way to interrogate both signaling molecules in real-time in living cells. Dual-FRET data presented here show that cAMP and Caspase-3 activation are inversely correlated during oxidative stress and hyperglycemia in living cells. These data are consistent with previous biochemistry results demonstrating that oxidative stress decreases cAMP via NADPH oxidase production [18]. During hyperglycemia alone, we show that cAMP and Caspase-3 are both increased over low-glucose controls, which confirms a redox-state dependent relationship between these pathways.

## Supporting information

S1 Fig Dual-FRET experimental averages. Time course of Caspase-3 activation (dotted lines) and cAMP (solid lines) after stimulation with (A) IBMX/forskolin (B) STS (C) 20 mM glucose (D) 20 mM glucose with H2O2, normalized to 5 mM glucose controls. These data represent averages with standard deviations from cells in 3 different dishes and each dish had N = 4–20 cells.
(TIF)
