# Supplementary figures and images for: Hyperspectral imaging for simultaneous measurements of two FRET biosensors in pancreatic β-cells
Source: PLoS One. 2017 Dec 6;12(12):e0188789. doi: 10.1371/journal.pone.0188789 (PMC5718502; doi:10.1371/journal.pone.0188789)

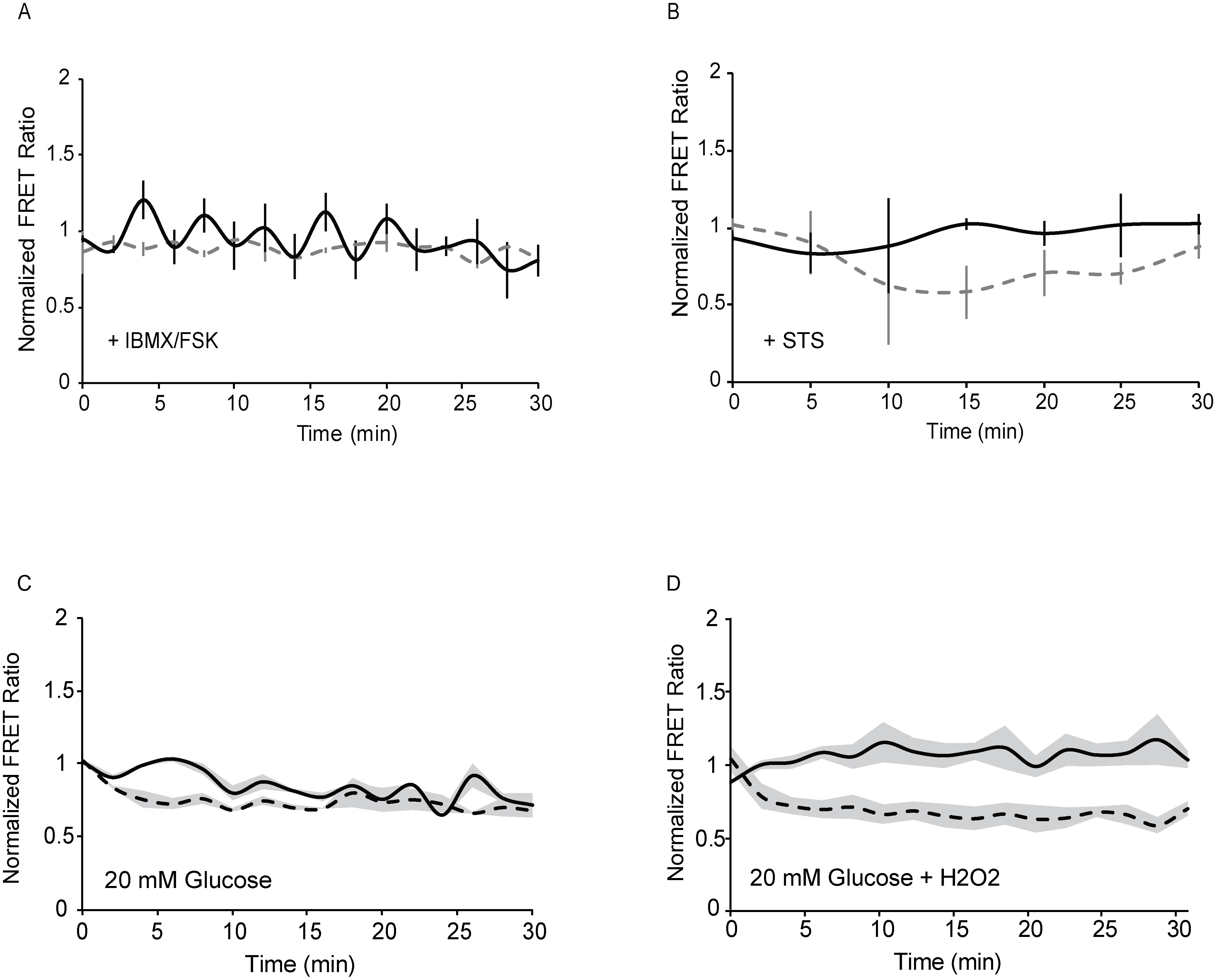

Supplement: S1 Fig — Time course of Caspase-3 activation (dotted lines) and cAMP (solid lines) after stimulation with (A) IBMX/forskolin (B) STS (C) 20 mM glucose (D) 20 mM glucose with H2O2, normalized to 5 mM glucose controls. These data represent averages with standard deviations from cells in 3 different dishes and each dish had N = 4–20 cells. (TIF) [file pone.0188789.s001.tif]
